# A MOMENT’S NOTICE: RECOGNIZING THE STRESSFUL LIFE EVENTS, EMOTIONS, AND ACTIONS THAT MAKE US SUSCEPTIBLE TO SCAMS

**DOI:** 10.1093/geroni/igac059.2289

**Published:** 2022-12-20

**Authors:** Alicia Williams, Lona Choi-Allum, Doug Shadel, Karla Pak, Chuck Raineville

**Affiliations:** AARP, Washington, District of Columbia, United States; AARP, Washington, District of Columbia, United States; AARP, Seattle, Washington, United States; AARP, Seattle, Washington, United States; AARP, Washington, District of Columbia, United States

## Abstract

Scamming has become a booming, multibillion-dollar industry with millions of Americans targeted by scams each year. To better understand the rates of exposure to scams and factors that may increase a target’s susceptibility to financial loss resulting from a scam, AARP commissioned a national survey that screened over 9,000 adults ages 18+ about their rates of encounters and financial loss from 26 popular scams. Among the total sample, 15% (an estimated 33 million people) reported losing money to a scam in the past year. Additionally, a subset of 3,280 respondents, including 1,085 respondents who lost money to a scam, were surveyed more extensively about their experiences. The study uncovered environmental and emotional factors in which targets are more susceptible to fraud. Specifically, fraud victims reported experiencing twice as many stressful life events (i.e., 4.0 vs. 1.9), 58% more fraud encounters, stronger emotional arousal during fraud encounters, and significantly less family support and closeness than non-victims. Additionally, only two of the eight protection tools recommended by fraud experts (i.e., using a passcode and installing protective software on one’s computer) were used by most respondents; and less than half of respondents reported using call blocking tools or online monitoring of their accounts, which can dramatically reduce exposure to scam encounters. Together, these findings show the importance of building public awareness of the role of stress, emotional arousal, and feelings of isolation in fraud susceptibility, and highlight the benefits of using fraud protection tools to limit one’s exposure to fraud.

